# Calculus

**Published:** 1861-09

**Authors:** 


### The Clinique.

### CHICAGO MEDICAL DISPENSARY.

**Service, Prof, E. ANDREWS, Attending Surgeon.**

Reported for the EXAMINER.

I. *Calculus.*—GENTLEMEN:—The patient before us this morning is to be sounded, for the purpose of ascertaining the presence or absence of a calculus in the bladder. Calculus is a disease which is fortunately quite rare in this vicinity. In certain sections of the country, which appear to be those where the spring-water is most saturated with lime and magnesia, the cases are said to be very numerous. Thus, in Kentucky and Tennessee, great numbers of cases have been reported. In this city, owing, probably, to the fact, that the water of the lake, which is our drink, is almost free from mineral constituents, calculus is so rare, that I have only seen three cases which originated strictly within our borders, and those three were all of them persons who lived on the outskirts of the city, and drank the highly mineralized water of the wells. Of cases found among those who use the lake water, I think it will be discovered in almost every instance, that they had their origin during a residence in some other locality.

A calculus once formed, goes on increasing in size, until, by the irritation caused by its presence as a foreign body, it inflames the bladder, destroys the health, and ultimately produces death. It is of the utmost importance, therefore, that the case be early diagnosed, and the removal of the calculus effected. I do not propose this morning to give the etiology and pathology of the disease, but simply to instruct you in the practical matters of the diagnosis.

The symptoms of a vesical calculus are rational and physical. The former consist of the effects produced on the patient by the presence of the object; the latter of the sounds and sensations which are communicated by contact with an instrument.

The key of the rational symptoms is found by considering that a rough foreign body resting on the delicate lining of the bladder, must cause chronic inflammation. This inflammation is aggravated by everything which causes the stone to shake and roll about, as riding, running, etc. Frequently on such occasions, it becomes severe and acute, and may be accompanied with a little discharge of blood. After the acute inflammation has subsided, the patient is much improved, but still has a chronic irritation, which lasts all through the intervals between his acute attacks. This inflammation differs from the mild chronic irritation of the prostrate common among young men, in being generally aggravated by shaking and jolting.

Some tenderness is often felt over the pubis. If the cause of the trouble is not removed, all the symptoms become worse. The inflammation is more continuous and severe; it extends often up the ureters to the kidneys, involving them in permanent disease; the urine is ropy and cloudy, the pain is great, and the exhaustion ultimately fatal. Very early in the disease, one curious symptom generally presents itself, and that is a pain referred by the patient to the glans penis. If the organ be examined, it will be found free from inflammation, showing that the suffering is sympathetic, merely, being referred to the wrong spot, exactly as the pain of the knee is in hip disease. The suffering is such, that the patient often tries to alleviate it by squeezing the glans.

Half-way between the rational and the physical symptoms may be placed the effects of the calculus on micturition. The patient often finds that the urine, after commencing to flow in a full stream, is suddenly checked by the calculus rolling upon the neck of the bladder, and acting as a valve to the orifice; but after lying down, or shifting in some way the position of the stone, he is enabled to recommence the urination. This symptom is not always present. I have operated upon one severe case, where it had never existed, owing to the calculus being half encysted, so that it was fixed in its position, and did not roll about.

Some patients suffer from incontinence of urine, and in fact, almost the whole catalogue of urinary inconveniences might be added to the list of occasional symptoms.

When several of these signs are distinctly present, you can often make almost a certain diagnosis from the rational symptoms alone, but still there is no absolutely conclusive evidence of the calculus, except, to feel it with the sound, and we will now proceed to apply the test:

The instrument which you here see, is made of polished steel, and is of the exact shape of a catheter, save that it is solid instead of hollow. Indeed, by corking up the extremity of a silver catheter, you may convert it into a perfectly good sound. As the parts around the bladder and urethra are very sore and tender, and as it is important to have perfect quietness for the purpose of a thorough exploration, I have directed the patient be anæsthetized before being brought in. This being accomplished, I will now introduce a catheter, and fill the bladder with water. This is advisable, because the cavity being thus enlarged, your instrument moves about more freely, and enables you to judge better of the size of the stone. Having moderately distended the viscus, I proceed to introduce the sound exactly as I would a catheter. It has now entered the bladder, and I feel the peculiar gritty sensation of a calculus. If you listen carefully while I strike the instrument against it, you will hear a distinct click as of steel against a hard substance. When the calculus is quite small, this clicking sound is very faint, and can only be heard by putting the ear close to the handle. And here, gentlemen, let me give you a caution about instruments. I have seen sounds made with the handle formed of a separate piece, screwed upon the shaft. Such handles are liable to work loose, and to give a false click themselves. The handle and shaft should be of one piece. To judge of the size of this stone, I move the end of the instrument across it in various directions, and by the amount of motion, endeavor to estimate the diameter. I also put the instrument against it, and toss it about, and thus try to judge of its weight and momentum. For this latter test, a corked silver catheter is better than a solid sound, because, having little weight or momentum of its own, it transmits more clearly to the fingers of the operator, the shock of contact with the calculus. In the present instance, I judge the stone to be a little over an inch in diameter, a size rather above the average, but by no means as great as some which I have seen.

Sometimes a stone is lodged in a sacculated cavity of the walls of the bladder, and it is then said to be encysted. If one extremity projects into the general hollow of the viscus, there is no difficulty in feeling it with the sound; but if it is lodged so deeply as to be covered by folds of membrane, you may not be able to find the orifice of the sacculation, and may thus fail to detect the object. In such cases great care should be exercised to carry the instrument over every part of the vesical walls, and the sounding may be repeated in different positions several times. After all, in rare instances, there may remain a concealed calculus, but as a general rule, if, after two careful and thorough explorations you find nothing, you will be safe in asserting that no calculus is present.

The operation of sounding, is, of course, irritating to the organs; hence, the patient must, after it is over, be directed to keep quiet for a day or two, and if requisite, make use of measures adapted to prevent the occurrence of cystitis.

In the present case, I shall recommend the removal of the stone by operation, and at the time of its performance, will give you full instructions upon that topic.

